# Utilizing Contrast-Enhanced Computed Tomography for Accurate Localization of an Omental Lymphangioma: A Case Report

**DOI:** 10.7759/cureus.77532

**Published:** 2025-01-16

**Authors:** Takayuki Fujii, Aya Tanaka, Hiroto Katami, Ryuichi Shimono

**Affiliations:** 1 Pediatric Surgery, Kagawa University Faculty of Medicine, Kita-gun, JPN; 2 Pediatric Surgery, Kagawa University, Takamatsu, JPN

**Keywords:** acute abdomen, computed tomography, cystic lymphangioma, imaging modalities, intra-abdominal mass, laparoscopic-assisted resection, lymphatic malformation, omental lymphangioma, pediatric abdominal tumors

## Abstract

Omental lymphangioma, a rare congenital benign lesion, represents a small proportion of abdominal lymphangiomas. Its diagnosis can be challenging, owing to its nonspecific symptoms and features that overlap with those of other cystic abdominal conditions.

The report presents a case of an eight-year-old girl with progressive abdominal distension noted since the age of two years. Initially misattributed to constipation, the significantly worsening distension was later evaluated through imaging studies. Ultrasonography, magnetic resonance imaging (MRI), and contrast-enhanced computed tomography (CT) revealed a massive cystic lesion occupying the abdominal cavity. Although ultrasonography and MRI suggested a cystic mass, only contrast-enhanced CT could identify vessels within the septa as being branches of the gastroduodenal artery, thereby confirming the origin of the lesion to be in the greater omentum. The patient underwent laparoscopic-assisted omentectomy, with the aid of a SAND balloon catheter (Hakko Medical Industry, Tokyo, Japan), to prevent fluid leakage during the aspiration of 4.7 L of cystic fluid. Pathological examination confirmed the diagnosis of cystic lymphangioma. No recurrence has been observed over a follow-up period of more than five years. This case highlights the critical role of contrast-enhanced CT in localizing large abdominal lymphangiomas based on vascular anatomy and demonstrates its importance in guiding the surgical planning and management of this disease.

## Introduction

Omental lymphangioma is a benign abdominal condition that primarily affects children [1]. Cystic lymphangiomas are most commonly found in the cervical region and mediastinum, whereas abdominal lymphangiomas are relatively rare, accounting for approximately 3% to 9.2% of lymphangioma cases [2,3]. Symptoms typically include abdominal distension, which may be accompanied by a palpable mass or pain [1,4]. Diagnosis can be challenging, often requiring multiple imaging modalities [4,5]. The report presents a case of a patient with a large omental lymphangioma, in which contrast-enhanced computed tomography (CT) was effective in localizing the lesion.

## Case presentation

**Figure 1:**
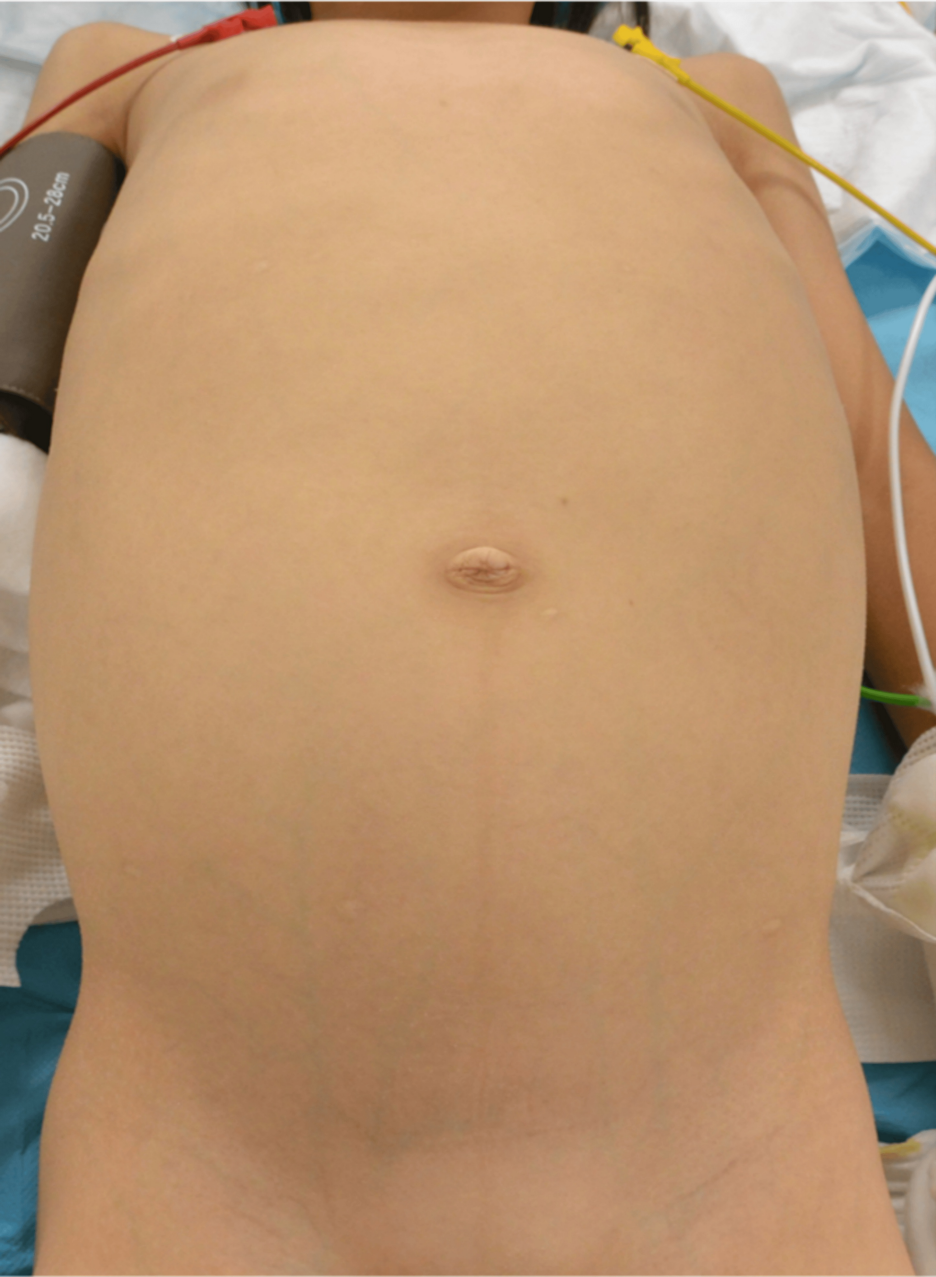
Findings on the external appearance Significant abdominal distention is noted.

An eight-year-old girl presented with abdominal distension that had been noticed since she was two years of age but was initially attributed to constipation. The patient had a history of umbilical hernia, diagnosed at the age of two years. The abdominal distension was further noted during a school health check-up but remained untreated. The patient sought medical attention following an episode of upper respiratory symptoms, during which significant abdominal distension was observed by a local physician. Subsequent abdominal CT revealed a cystic mass, prompting referral to our hospital (Figure 1).

The C-reactive protein level was mildly elevated at 1.0 mg/dL. Only minor elevations of carbohydrate antigen 125 (45 U/mL), and neuron-specific enolase (16.7 ng/mL) were observed (Table 1).

**Table 1:** Tumor markers of the patient

Laboratory parameters	Value	Reference ranges
Alpha-fetoprotein (AFP)	1.0	10 (ng/mL)
Carbohydrate antigen 125 (CA125)	45	35 (U/mL)
Neuron-specific enolase (NSE)	16.7	13 (ng/mL)
Vanillylmandelic acid (VMA)	3.7	6-11 (µg/mg Cre)
Homovanillic acid (HVA)	6.2	11-20 (µg/mg Cre)
Serum beta human chorionic gonadotropin (β-hCG)	0.1	0.1 (ng/mL)
Interleukin 2 receptor (IL-2-R)	427	121-613 (U/mL)

**Figure 2:**
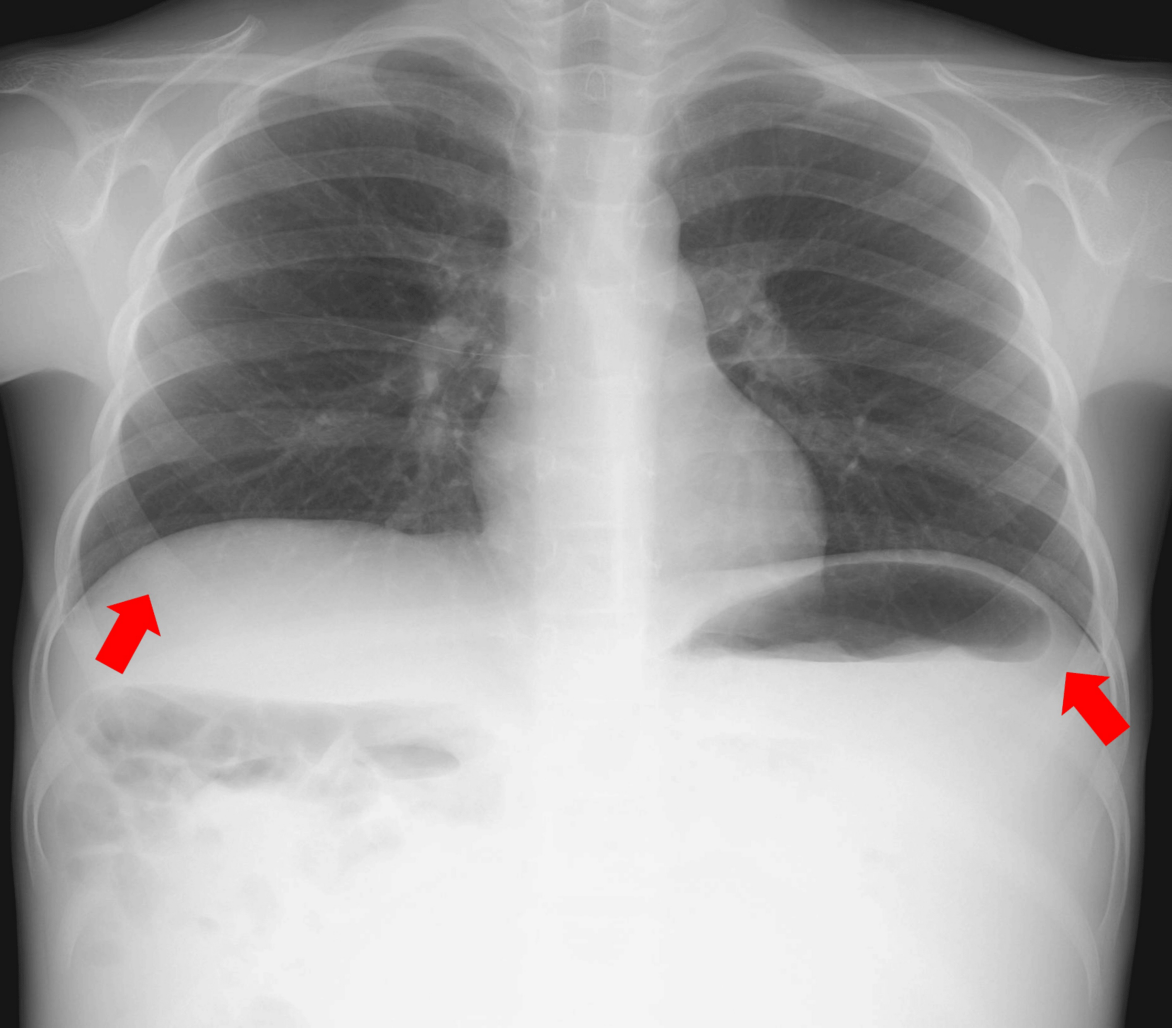
Chest X-ray (posterior-anterior) Diaphragmatic elevation is evident (red arrows).

**Figure 3:**
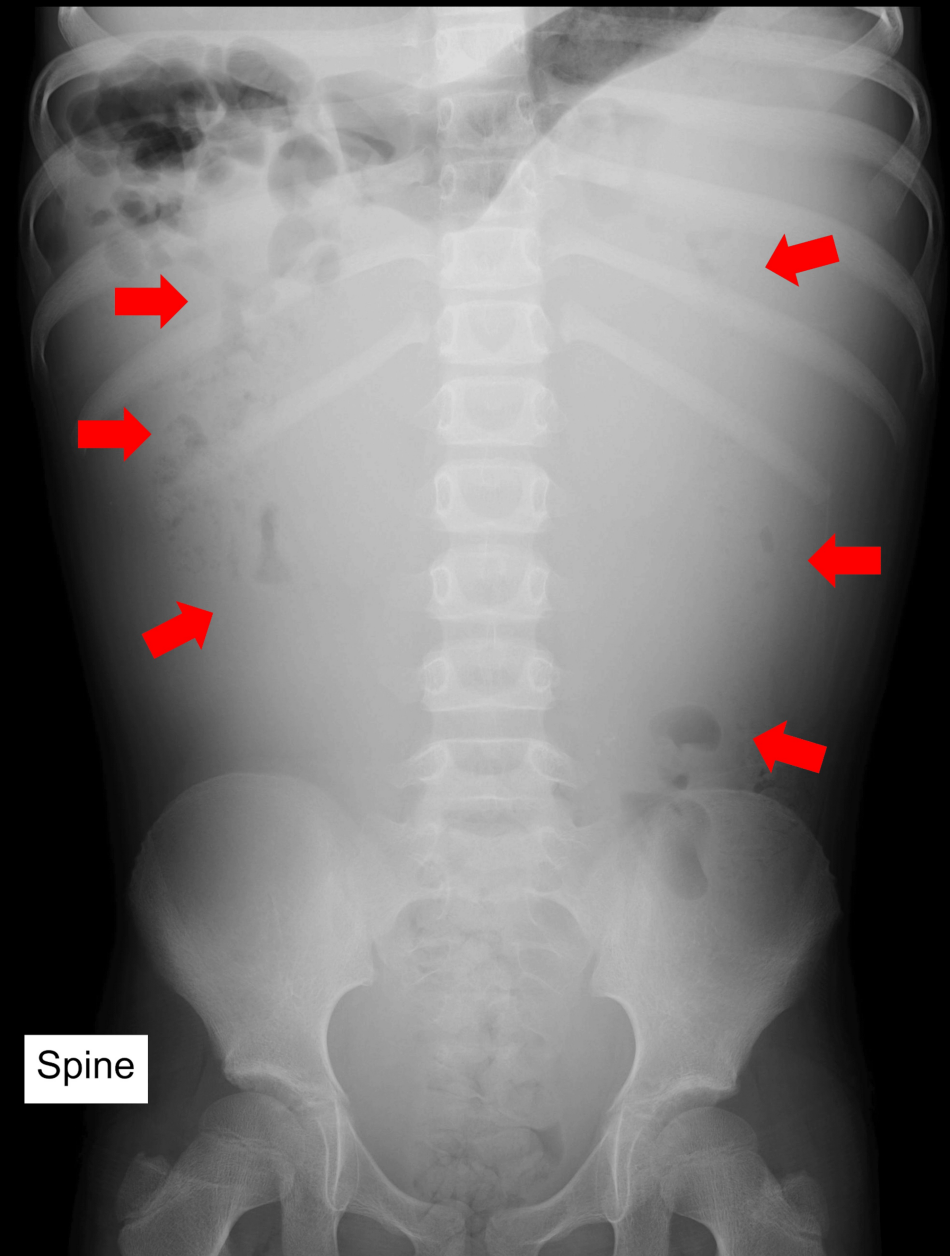
Abdominal X-ray (anterior-posterior) Displacement of the bowel loops by an abdominal mass is evident (red arrows).

A chest and abdominal X-ray demonstrated diaphragmatic elevation and displacement of the bowel loops by an abdominal mass (Figures 2-3).

**Figure 4:**
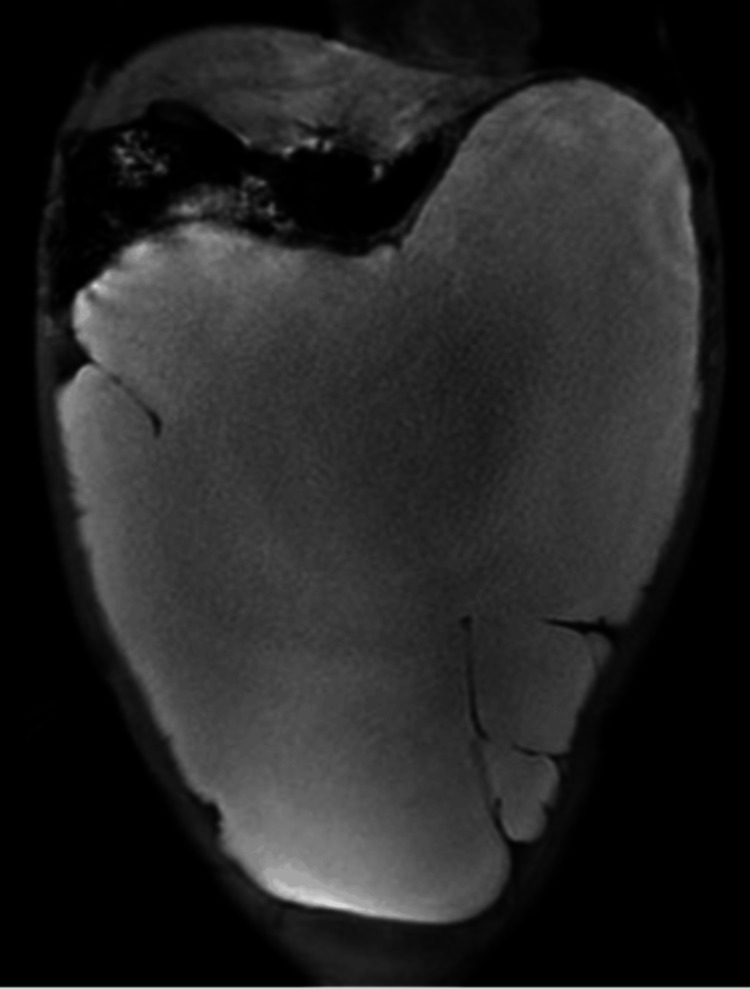
Magnetic resonance imaging (coronal view, T2-weighted) A well-defined cystic mass measuring 30 cm in diameter with high signal intensity is shown.

A T2-weighted magnetic resonance imaging (MRI) showed a well-defined cystic mass measuring 30 cm in diameter, with high signal intensity (Figure 4).

**Figure 5:**
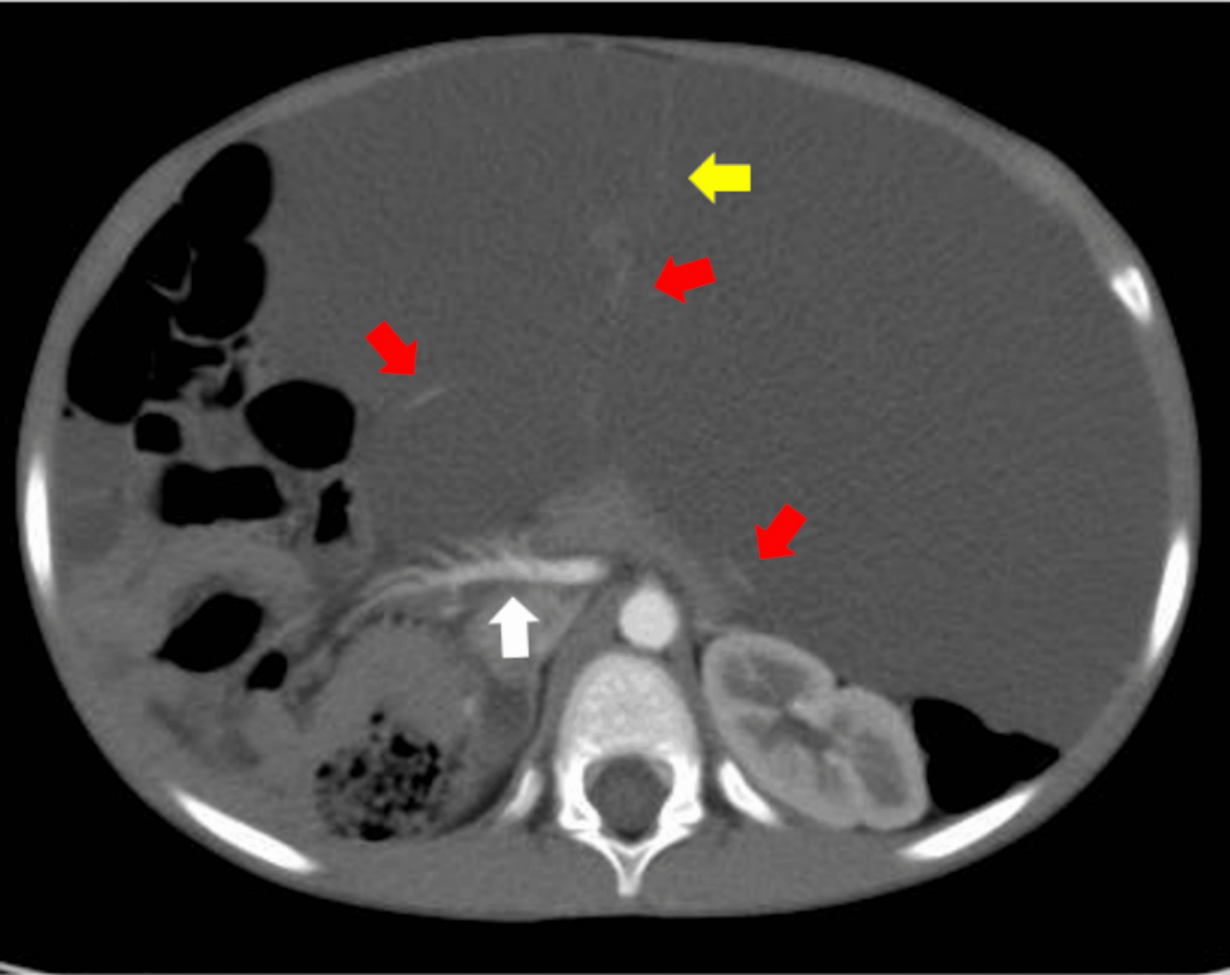
Contrast-enhanced abdominal computed tomography (axial view) A massive fluid-density cystic lesion occupying the abdominal cavity was revealed. The lesion's septations (yellow arrow) and vessels running within the septa were branches of the gastroepiploic artery (red arrows), confirming the greater omentum as the cyst's origin. The superior mesenteric artery was displaced to the right (white arrow).

Contrast-enhanced abdominal CT revealed a massive fluid density cystic lesion occupying the abdominal cavity. The lesion's septations and vessels running within the septa were branches of the gastroepiploic artery, confirming the greater omentum as the cyst's origin. The superior mesenteric artery was displaced to the right (Figure 5).

**Figure 6:**
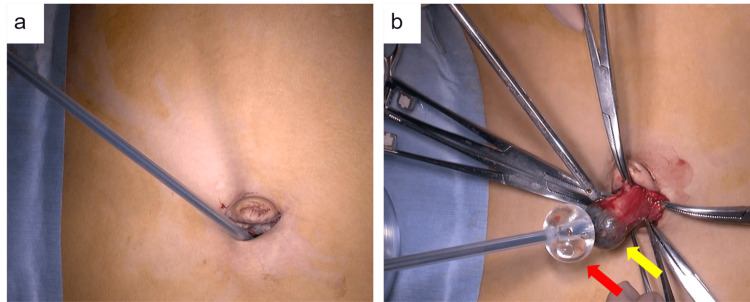
SAND balloon catheter (a) Aspiration of cystic fluid using a SAND balloon catheter (Hakko Medical Industry); (b) Prevention of fluid dispersion by inflating the balloon inside (yellow arrow) and outside (red arrow) the cyst wall.

The patient underwent a laparoscopic-assisted omentectomy. A lower umbilical incision was created to access the abdominal cavity, following which a SAND balloon catheter (Hakko Medical Industry, Tokyo, Japan), with inflation both inside and outside the cyst wall, was used to prevent fluid leakage during aspiration (Figure 6).

**Figure 7:**
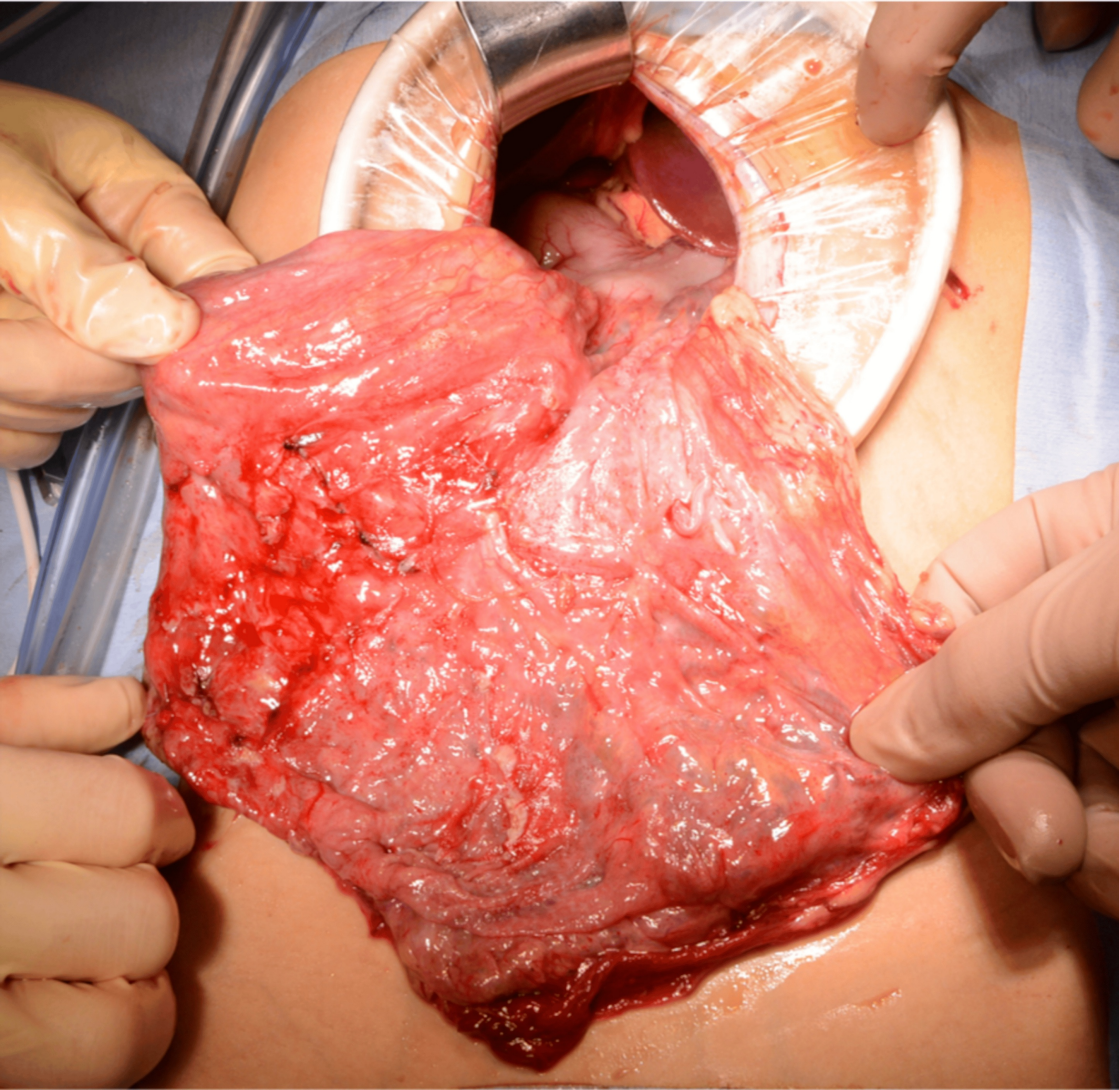
Intraoperative findings Perioperative examination showing a lymphangioma originating from the greater omentum.

In total, 4.7 L of cystic fluid was aspirated. Significant adhesions were observed within the abdominal cavity, necessitating the placement of 5-mm ports in the left upper and lower quadrants. Dissection was performed using an electrocautery device and scissors. We confirmed the lesion to be a lymphangioma originating from the greater omentum (Figure 7).

Owing to the substantial size of the mass, the incision was extended 4.5 cm from the umbilicus to the upper abdomen, facilitating exteriorization and complete resection of the lesion. Pathological examination confirmed the diagnosis of a cystic lymphangioma. No recurrence has been observed during a follow-up period of over five years postoperatively.

## Discussion

An intra-abdominal lymphangioma is a rare congenital benign lesion, occurring in approximately 1 in 27,000 pediatric patients admitted to hospitals [6]. Although hemorrhage and abdominal symptoms may occur, the absence of other significant symptoms is the reason that some patients are diagnosed only after the lesion has reached a substantial size [4,7,8]. Although the exact duration of the condition in this particular patient remains unclear, it is likely to have been a prolonged process, as abdominal distension and an umbilical hernia were noted as early as two years of age.

Diagnosis can be challenging, often requiring multiple imaging modalities [4,5]. Although ultrasonography can identify cystic lesions, determining the primary origin can be challenging in patients with large masses. Additionally, differentiating such lesions from ascites can be difficult [9]. MRI has shown superiority in diagnosing large hemorrhagic cystic masses and differentiating them from free intraperitoneal fluid [10]. For this patient, however, the MRI did not provide sufficient information for the localization of the lesion. Conversely, contrast-enhanced CT revealed that all the vessels within the septa of the cystic lesion were branches of the gastroduodenal artery, enabling a diagnosis of greater omental origin. To date, there have been very few reports of cases in which contrast-enhanced CT allowed for the localization of a large intra-abdominal lymphangioma based on vascular anatomy [5]. Despite the issue of radiation exposure, CT may be a valuable tool for localizing large intra-abdominal lymphangiomas and guiding the choice of surgical approach.

Surgical excision is the preferred treatment, with complete resection resulting in excellent prognosis and low recurrence rates [7]. In some patients, laparoscopic or laparoscopic-assisted excision has been successfully employed [11,12]. A SAND balloon catheter was used during surgery to prevent leakage of the cystic fluid. Commonly used in ovarian cyst surgeries, this technique involves sandwiching the cyst between two balloons - one inside and one outside the cyst wall - to prevent spillage and contamination. The use of SAND balloons to manage potentially malignant and complex renal cysts has been reported [13].

## Conclusions

Omental lymphangioma is a rare but significant differential diagnosis for intra-abdominal cystic masses in children. This case demonstrates the critical role of contrast-enhanced CT in localizing the lesion and confirming its origin based on vascular anatomy. These findings highlight the importance of accurate imaging and diagnosis in managing patients with large omental lymphangiomas.
